# High-sensitivity deuterium metabolic MRI differentiates acute pancreatitis from pancreatic cancers in murine models

**DOI:** 10.1038/s41598-023-47301-7

**Published:** 2023-11-15

**Authors:** Elton T. Montrazi, Keren Sasson, Lilach Agemy, Dana C. Peters, Ori Brenner, Avigdor Scherz, Lucio Frydman

**Affiliations:** 1 Department of Chemical and Biological Physics, Weizmann Institute of Science, Rehovot, Israel; 2 Department of Plant and Environmental Science, Weizmann Institute of Science, Rehovot, Israel; 3 Department of Radiology and Biomedical Imaging, Yale School of Medicine, New Haven, USA; 4 Department of Veterinary Resources, Weizmann Institute of Science, Rehovot, Israel

**Keywords:** Animal disease models, Cancer metabolism, Cancer screening, Magnetic resonance imaging

## Abstract

Deuterium metabolic imaging (DMI) is a promising tool for investigating a tumor’s biology, and eventually contribute in cancer diagnosis and prognosis. In DMI, [6,6′-^2^H_2_]-glucose is taken up and metabolized by different tissues, resulting in the formation of HDO but also in an enhanced formation of [3,3′-^2^H_2_]-lactate at the tumor site as a result of the Warburg effect. Recent studies have shown DMI’s suitability to highlight pancreatic cancer in murine models by [3,3′-^2^H_2_]-lactate formation; an important question is whether DMI can also differentiate between these tumors and pancreatitis. This differentiation is critical, as these two diseases are hard to distinguish today radiologically, but have very different prognoses requiring distinctive treatments. Recent studies have shown the limitations that hyperpolarized MRI faces when trying to distinguish these pancreatic diseases by monitoring the [1-^13^C_1_]-pyruvate→[1-^13^C_1_]-lactate conversion. In this work, we explore DMI’s capability to achieve such differentiation. Initial tests used a multi-echo (ME) SSFP sequence, to identify any metabolic differences between tumor and acute pancreatitis models that had been previously elicited very similar [1-^13^C_1_]-pyruvate→[1-^13^C_1_]-lactate conversion rates. Although ME-SSFP provides approximately 5 times greater signal-to-noise ratio (SNR) than the standard chemical shift imaging (CSI) experiment used in DMI, no lactate signal was observed in the pancreatitis model. To enhance lactate sensitivity further, we developed a new, weighted-average, CSI-SSFP approach for DMI. Weighted-average CSI-SSFP improved DMI’s SNR by another factor of 4 over ME-SSFP—a sensitivity enhancement that sufficed to evidence natural abundance ^2^H fat in abdominal images, something that had escaped the previous approaches even at ultrahigh (15.2 T) MRI fields. Despite these efforts to enhance DMI’s sensitivity, no lactate signal could be detected in acute pancreatitis models (n = 10; [3,3′-^2^H_2_]-lactate limit of detection < 100 µM; 15.2 T). This leads to the conclusion that pancreatic tumors and acute pancreatitis may be clearly distinguished by DMI, based on their different abilities to generate deuterated lactate.

## Introduction

Distinguishing between pancreatitis and pancreatic cancer is a crucial step in the prognosis and management associated with both diseases. However, identifying the precise diagnosis of the two ailments can be challenging, as their clinical and radiological features often overlap significantly^1,2^. The most widely used pancreatic imaging methods, including CT and ultrasound, can confound adenocarcinomas with non-lethal cysts, pancreatitis, and other kinds of inflammations that provide similar contrasts in these exams. This may lead to repeated biopsies of suspicious radiological findings. Metabolic processes can also be similar, as inflammations may show enhanced glucose uptake, and hence lead to increased FDG-PET signatures in both cases^3^. In view of this several studies have explored potential methods based on MRI, to differentiate between pancreatitis and pancreatic cancer. These include MR cholangiopancreatography (MRCP), which is often used delineate the pancreatic duct system and can detect cancers not easily visualized by CT^4^, ^1^H-based Magnetic Resonance Spectroscopy (MRS^5,6^) and—more recently—hyperpolarized ^13^C Magnetic Resonance Spectroscopic Imaging (MRSI)^7–9^. The latter in particular has been used to monitor the conversion of hyperpolarized [1-^13^C_1_]-pyruvate into metabolic products like [1-^13^C_1_]-lactate, [1-^13^C_1_]-alanine, and ^13^C-bicarbonate^10^. This technique has revealed significant increases in lactate production in both mice models of pancreatic tumors and in human patients. The approach, however, has also revealed that sizable amounts of [1-^13^C_1_]-lactate form out of [1-^13^C_1_]-pyruvate in pancreatitis mice models, in proportions comparable to the concentrations seen for small and mid-size pancreatic tumors^8,9^. This in turn, complicates the prospects of this approach for differential diagnoses.

Inspired by ^13^C metabolic MRSI, recent years have witnessed the introduction of ^2^H-labeled precursors as potential cancer-oriented metabolic reporters. In the ensuing Deuterium Metabolic Imaging (DMI) experiment, [6,6′-^2^H_2_]-glucose is provided by injection or gavaging into an animal or patient, and the ensuing formation of [3,3′-^2^H_2_]-lactate as result of the Warburg effect is monitored^11–18^. Mapping these resonances as well as the ^2^H-water (HDO) that is metabolically generated can thus highlight tumors, as has been recently shown in pancreatic cancer assessments on mice^17,18^. In the present work, we test the capability of DMI to distinguish between pancreatic ductal adenocarcinomas (PDAC) and pancreatitis. These tests were initially conducted using multi-echo balanced SSFP (ME-SSFP) approaches, for which recent findings demonstrated ca 3–8 times signal-to-noise ratio (SNR) improvements over DMI data collected by conventional chemical shift imaging (CSI) techniques^18,19^. While having no problems in imaging sub-mM [1-^13^C_1_]-lactate concentration levels in tumor models, these DMI experiments failed to detect ^2^H-labeled lactate in mice subject to chemically-induced acute pancreatitis. To enhance sensitivity to lactate production even further, we explored the utilization of new SSFP variants, based on a weighted average k-space acquisition of CSI-SSFP data^20^. Non-Fourier processing methods provided these CSI-SSFP by additional SNR improvements per unit scan time of ca. fourfold vs ME-SSFP. Even with these sensitivity improvements, no [3,3′-^2^H_2_]-lactate could be observed in an acute pancreatitis model. We hypothesize that this major difference against the results observed with the use of hyperpolarized ^13^C MRI, resides in the relative ease and speed with which pyruvate is converted into lactate by most tissues. *We therefore conclude that, at variance with most other MRI, CT and ultrasound imaging modalities, DMI opens a realistic window to the differentiation between pancreatic tumors and pancreatitis*.

## Methods

### Optimized DMI spectroscopic imaging sequences

**Figure 1:**
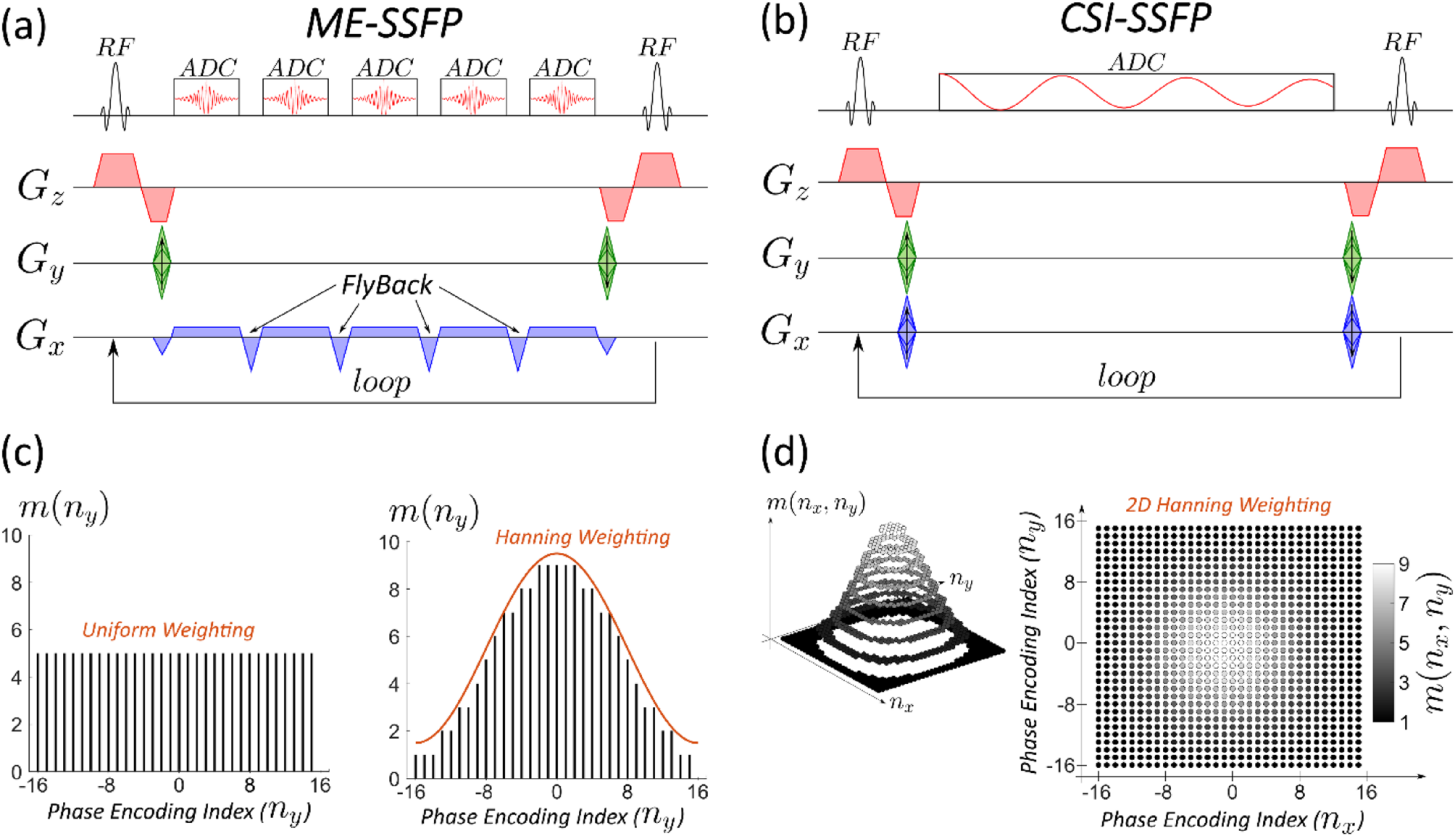
Top: Slice-selective ME-SSFP and CSI-SSFP sequences used in the study. These sequences’ 1D and 2D indirect-domain PE dimensions were sampled in a uniform way or weighted by the Hanning-like functions shown on the bottom. In all cases, 32 points were encoded in the in-plane domains.

The starting point of our experiments is the ME-SSFP sequence^18,21,22^, exemplified in Fig. 1a with five echoes and serving as a sensitivity-optimized platform for resolving the ^2^H MRI responses of deuterated water, glucose, and pyruvate. The experiment relies on balanced SSFP, with the oscillating readout gradient’s echo spacing and offset adjusted so as to match the a priori known chemical shifts of these three species^18,21^. Acquiring five echoes for separating these three species gives extra robustness to the least-square fitting protocol used to perform the data analysis^23,24^, as well as an ability to incorporate field inhomogeneities –the latter aided by the use of periodically collected ^1^H-based abdominal field maps^19^. The ME-SSFP sequence also controlled the readout bandwidth, voxel size, and field of views (FOV) of the in-plane imaging domains; the remaining dimension was defined by a slice-selective pulse. Since processing ME-SSFP’s odd and even echoes jointly introduced data inconsistencies, fly-back gradients were used, and only odd echoes were processed (Fig. 1a)^18^. As the ensuing data discarding reduces the sequence’s efficiency, the CSI-SSFP variant illustrated in Fig. 1b, was also assayed^20^. CSI-SSFP does not use a readout gradient and so its spatial encoding needs to incorporate an additional phase encoding (PE) loop; on the other hand, the Free Induction Decay (FID) acquisition is more efficient, and can be set to achieve the same steady-state transverse magnetization as ME-SSFP. This enhanced efficiency offsets the penalty for the additional PE dimension, as multiple scans—be them in the form of signal averaging or of multiple phase-encodes—are required by DMI anyhow for the sake of achieving sufficient sensitivity. The SNR of these experiments was enhanced further by the use of weighted average acquisitions (Fig. 1c and d)^25–30^; in ME-SSFP this could be performed solely along one PE dimension, whereas in CSI-SSFP this was accomplished along two directions. More details about the SNR improvements introduced by these weighted average methods, is given in the Supporting Information.

### Data processing

ME-SSFP generates a series of images associated to different echo times. Each voxel in these echo images undergoes amplitude and phase modulations, due to the various metabolic chemical shifts contributing to a given voxel. In practice, this spectroscopic signal corresponds to a FID that has been truncated so as to fulfill the SSFP optimal SNR conditions, with a number of data points equal to the number of echoes. These echo images were converted into chemical shift images using the "iterative decomposition of water and fat with echo asymmetry and least squares estimation" (IDEAL) protocol^23,24^, modified as described in our previous work^19^. In the CSI-SSFP experiments, a similarly truncated FID was acquired for each voxel, but with the dwell time and number of data points defined by the user. The first four points of this FID had to be discarded due to the digital receiver’s filtering. Chemical shift images were then obtained from the resulting FID using the IDEAL fitting method aided by ^1^H-based field mappings. Note that additional refinements incorporating fits along the kinetic dimension and denoising techniques^19^, could also be directly applied to these CSI-SSFP acquisitions.

## Experimental

### Phantom

Phantom tests were done on three tubes with enriched [6,6′-^2^H_2_]-glucose, [3,3′,3″-^2^H_2_]-lactate (both from Cortecnet, Voisins-le-Bretonneux, France), and ^2^H-water (Tzamal, Petach Tikva, Israel). These metabolites were dissolved in 5 mm tubes at approximately 50 mM ^2^H concentrations in 2% agarose in Dulbecco’s Phosphate Buffered Saline (PBS).

### In vivo procedures

All animal procedures were approved by the Institutional Animal Care and Use Committee of the Weizmann Institute of Science, which is fully accredited by the AAALAC, the US NIH Office of Laboratory Animal Welfare, and the Israel Ministry of Health. All methods and procedures were performed in accordance to relevant guidelines and regulations. This study is reported in accordance with ARRIVE (Animal Research: Reporting of In Vivo Experiments) guidelines.

#### Tumor model

Fifteen C57 black mice were implanted with KPC cells leading to a rodent-based pancreatic cancer^9^, and examined ca. two weeks after implantation. Seven of these were examined employing ME-SSFP, five were measured using CSI-SSFP, and three were used in ME-SSFP vs CSI-SSFP comparisons. KPC is a genetically engineered mouse model possessing key genetic similarities with human PDACs^31^, which have made it a common model for drug discovery purposes. KPC’s similarities with human PDAC include common immune defects, barriers to effector cell infiltration, and similar immune checkpoint signaling^32^.

#### Acute pancreatitis model

Ten C57 black mice were subjected to a series of caerulein injections, leading to acute mild pancreatitis symptoms^9,33^. In this protocol, mice are fasted 8 h before performing seven hourly intra-peritoneal injections of caerulein (Sigma, St. Louis, MO, USA) diluted in PBS (dose of 50 µg/kg of body weight); this procedure is then repeated 48 hs later. This protocol leads to mild acute pancreatitis symptoms—mild as in opposed to severe, and acute as in a time-limited, non-chronic reaction. An important feature that follows from the acuteness of the inflammation is the need to scan the animals shortly after the second batch of caerulein injections, as otherwise the symptoms may be gone. The mice were thus examined by DMI ca. 1 h after the last injection, which is when the maximum inflammatory effects arise. Out of these ten mice, five of them were measured by ME-SSFP, and five by CSI-SSFP.

#### Control group

A control group consisting of three C57 black mice was subject to the same protocol as for the acute pancreatitis model, but using solely the PBS solution injections. Two of the mice were examined using ME-SSFP, while the remaining one was studied using CSI-SSFP. An additional control C57 black mouse was used to assess the conversion of pyruvate to lactate by ^2^H MRS.

All mice were anesthetized with a 20/80 O_2_/N_2_ stream containing an additional 3% isoflurane to induce sedation, and ~ 1–2% to keep sedation during the MRI scan. For the DMI experiments, ~ 2 g/kg body weight of [6,6′-^2^H_2_]-glucose in PBS were injected via a tail-vein line. Mice were euthanized immediately at the conclusion of these scans by increasing the isoflurane to 5%, and their pancreatic tissues and tumors subject to H&E histological analyses.

### MR imaging

All ^2^H/^1^H measurements were performed on a 15.2 T Bruker scanner running Paravision 6, using 20 mm diameter surface coils tuned to 649.93 (^1^H) and 99.77 MHz (^2^H). ME-SSFP and CSI-SSFP sequences (Fig. 1 and available at https://www.weizmann.ac.il/chembiophys/Frydman_group/software) were written for this setup, and were optimized for DMI using the following parameters: 2 ppm carrier frequency, TR = 11.48 ms, flip angle = 60°, 32 × 32 matrices, in-plane FOV = 40 × 40 mm^2^, 20 mm slices accommodating most of the abdomen excited using a 0.63 ms long pulse. For ME-SSFP: five gradient echoes (TE = 2.1 ms), flybacks of 0.257 ms, 20 kHz receiver bandwidth. For CSI-SSFP a similar TR was used, with a 46-point, gradient-free FID sampled at 5 kHz (out of which the 4 initial points had to be discarded as they were corrupted by the digital filtering). Uniform and weighted signal averaging of the phase-encoding (PE) domains were applied, in all cases using 0.2 ms long gradient pulses. Uniform ME-SSFP and CSI-SSFP used 1024 and 32 repetitions for their single- and dual PE encodings, respectively. For the acquisition-weighted experiments, ME-SSFP used 512 repetitions with the weighting coefficient NA = 4 (see Supporting Information, Eq. (5)); weighted and CSI-SSFP experiments used 12 repetitions with NA = 8, or 6 repetitions with NA = 20 (Supporting Information, Eq. (6)). Signal averaging was thus ~ 6 min for both the uniform and weighted CSI-SSFP and ME-SSFP DMI acquisitions. ^1^H coronal images were collected for localization purposes using TurboRARE: 10 slices, 0.8 mm thickness, same FOVs as DMI, 512 × 512 encoding matrix. ^1^H B_0_ maps were obtained by 3D double gradient echo, with same FOVs as for DMI and 64 × 64 × 8 encoding matrices.

Spatial domains for both the ME-SSFP and CSI-SSFP experiments were reconstructed identically, by 2D Fourier transform after zero-filling to 64 × 64 points. Images arising from the separated ME-SSFP echoes and for each CSI-SSFP FID point (5 frames for ME-SSFP, and 42 for CSI-SSFP from 46 acquired points) were processed using the RK-SpecRecon (Regularized kinetics spectral reconstruction) algorithm^19^, which isolated the images of the individual sites using a priori known chemical shift positions (4.7, 3.6 and 1.2 ppm for the three DMI metabolites; 2 ppm carrier frequency) and regularization of the metabolites’ evolution along the kinetic series. ^1^H-based B_0_ maps were used as initial guesses in the fitting to avoid “swaps” otherwise observed upon using the IDEAL processing^19^. To translate the intensities into metabolic concentrations, ^2^H’s natural abundance (~ 10 mM concentration prior to injection in liquid-containing spaces), together with SSFP’s signal attenuations as expected from the scanning parameters and the T_1_/T_2_ for each species, were used as described in Ref.^18^.

## Results

**Figure 2:**
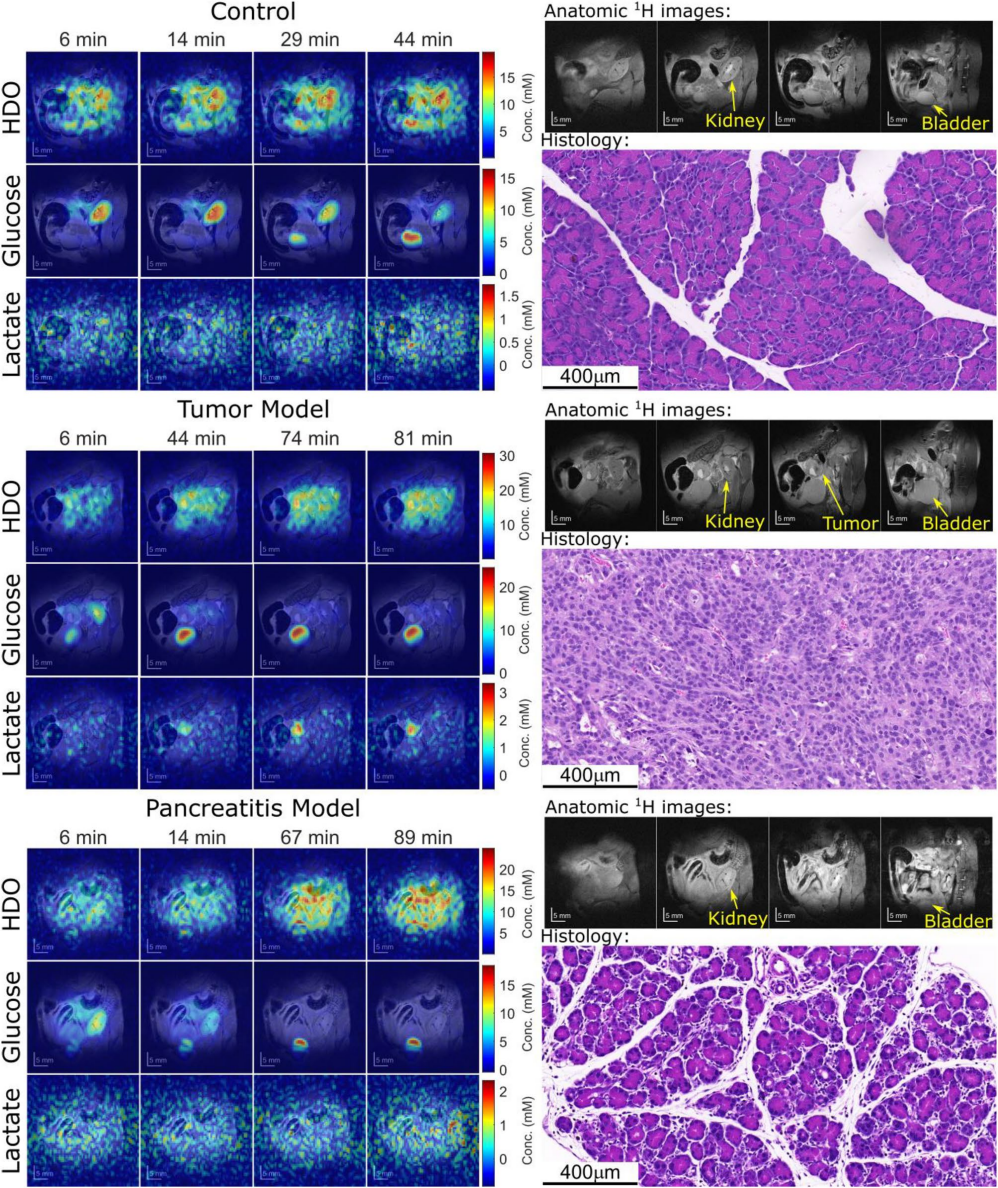
Right: Anatomic ^1^H images evidencing the main regions targeted by DMI and histological H&E slices arising for the pancreas for control, tumor, and pancreatitis models. Left: ^2^H ME-SSFP images as a function of time, following the injection of deuterated glucose. Concentrations were calculated as described in Ref.^18^. Histological sections on the right show normal pancreatic tissue (top), PDAC-induced differentiation (center), and induced pancreatitis as evidenced by edema and by inter- and intra-lobular infiltration by white blood cells. See the Supporting Information (Fig. S2) for additional histological evidence of the caerulein-induced inflammation introduced in other animals.

Figure 2 displays DMI images obtained using ME-SSFP, along with anatomical ^1^H images and histologies, for the control, KPC-implanted, and pancreatitis mice models. The anatomical images reveal the presence of the kidney, bladder, and tumor in the PDAC-implanted mouse. DMI images illustrate the time-dependent distributions of deuterated water, glucose, and lactate for all three mouse models. Note that immediately after injection glucose accumulates in the kidneys, but subsequently it gets distributed throughout the body while it gets consumed due to metabolism—and occasionally also via elimination into the bladder. In the case of the KPC-implanted mice, glucose clearly accumulates in the tumor region, mostly in a rim surrounding it. Furthermore, lactate is always clearly visible in the tumor region, at a concentration that is lower than that of glucose and in a location that is always within the tumor. Lactate levels peak around 45–60 min after the initial injection, and then diminish. By contrast, no ME-SSFP experiment revealed any lactate generation in either the control or the pancreatitis models (images not shown).

The absence of a lactate signal in the pancreatitis model prompted us to seek ways to enhance further the SNR of these DMI experiments. To do so, we relied on uniformly- and weighted-averaged CSI-SSFP acquisitions. While requiring a second PE loop, CSI-SSFP acquisitions should have—for the same number of overall scans—a higher sensitivity than ME-SSFP counterparts thanks to their avoidance of flyback gradient periods where no data are collected. Furthermore, both sequences offer the possibility of investing these scans in a uniform or a weighted fashion along their PE dimensions^25–30^, something that also may favor CSI-SSFP experiments possessing two such axes. Supporting Fig. S1 and Table 1 present ^2^H spectroscopic images and ensuing SNRs, arising from uniform/weighted CSI-SSFP and ME-SSFP experiments, obtained on a representative deuterated phantom containing water, glucose and lactate in separate compartments. Spectral separations were in all cases achieved with the same IDEAL processing. As can be appreciated from these data, the SNR improvement arising upon giving up the flyback gradients is ca. 10%, as this is approximately the time percentage that they occupied in the ME-SSFP acquisitions. On the other hand, the advantages of the weighted averaging are bigger, particularly in the CSI-SSFP experiment where it can be implemented along two axes (Table 1). These SNR improvements are ca. 1.7 for ME-SSFP and 2.5 for CSI-SSFP, while the theoretically expected values for the Hanning window weighting used are 2 and 4, respectively. These discrepancies reflect the fact that for the relatively small number of averages used (NA = 8 for CSI-SSFP, 4 for ME-SSFP) the Hanning window generated was imperfect. Moreover, notice that these improvements come at the cost of some blurring, which was noticeable in this well-defined tube-based phantom (Supporting Fig. S1) but not so in the in vivo experiments.

**Table 1:** Relative ratios between the SNRs afforded by various ^2^H MRSI approaches for a water/glucose/lactate DMI phantom.

	HDO	Glucose	Lactate
Uniform CSI-SSFP/uniform ME-SSFP	1.0 ± 0.2	0.9 ± 0.1	1.2 ± 0.1
Weighted ME-SSFP/uniform ME-SSFP	1.6 ± 0.3	1.6 ± 0.1	1.9 ± 0.1
Weighted CSI-SSFP/uniform ME-SSFP	2.4 ± 0.3	2.4 ± 0.2	3.0 ± 0.2
Weighted CSI-SSFP/uniform CSI-SSFP	2.4 ± 0.3	2.5 ± 0.3	2.6 ± 0.2

Errors reflect the statistics arising from n = 6 repeated experiments.

**Figure 3:**
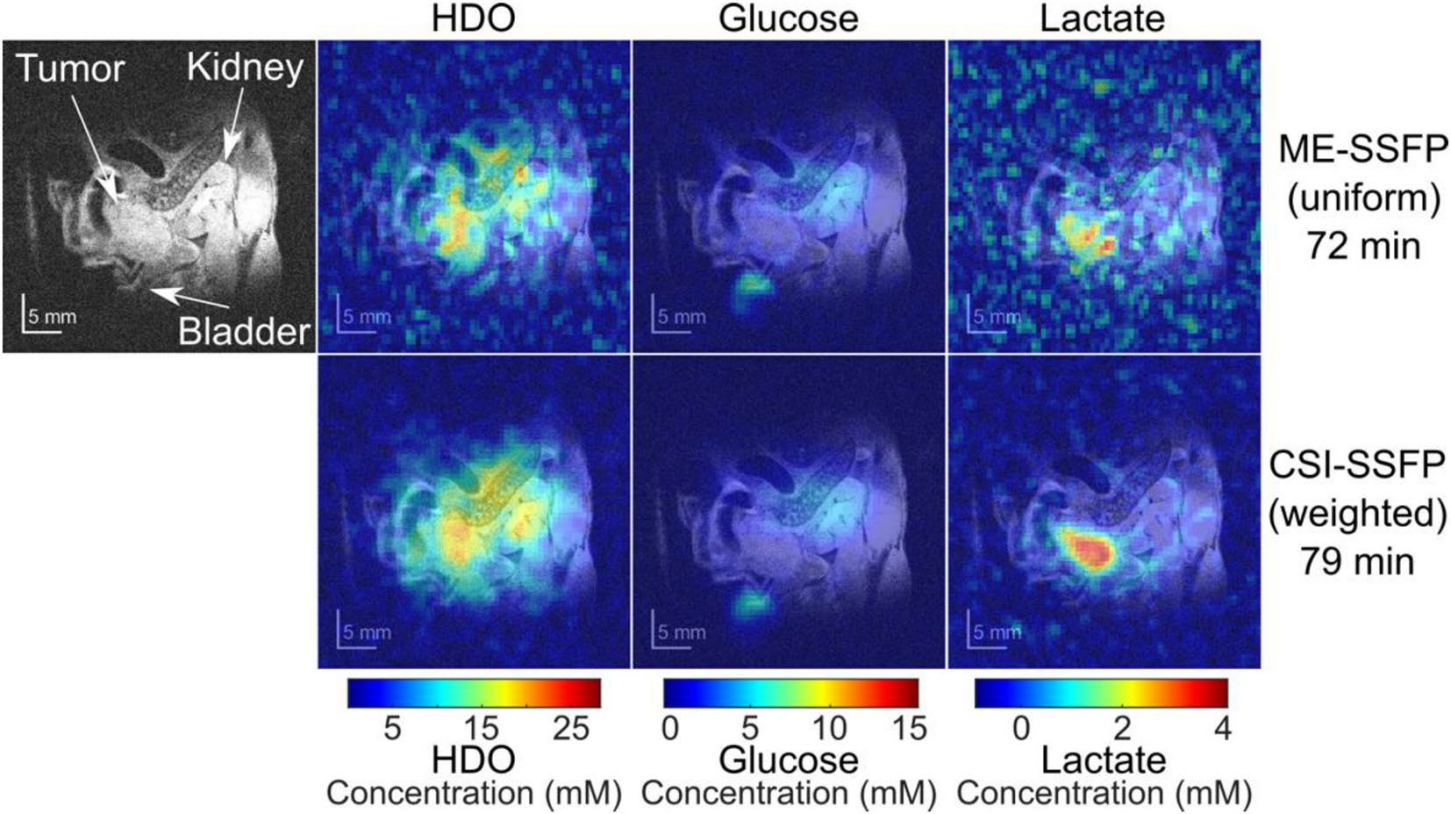
(Left) Anatomic ^1^H image of a PDAC-implanted mouse, showing among other tissues the kidney, bladder and tumor. (Right) DMI ME-SSFP (uniform sampling) and CSI-SSFP (weighted sampling) data collected at the indicated times after the injection of [6,6′-^2^H_2_]-glucose, shown overlaid on the ^1^H anatomic images. Lactate is present only in the tumor, HDO is spread throughout the body but also concentrates slightly inside the tumor, glucose is visible mostly in the bladder. Notice as well the superior SNR of the HDO and lactate CSI-SSFP images; “granularity” in the ME-SSFP images may be influenced by the higher noise in these experiments.

**Figure 4:**
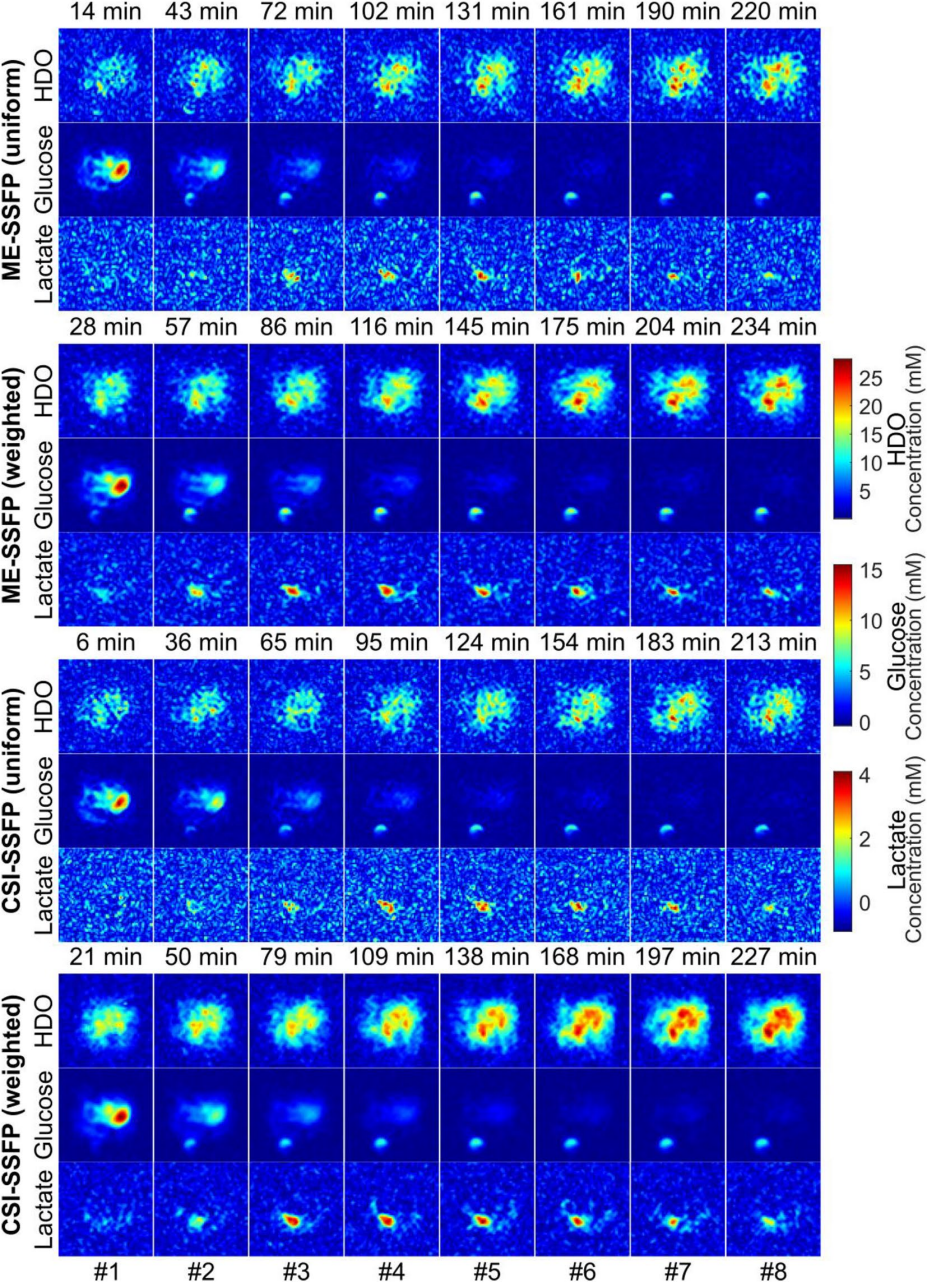
DMI images collected on the same PDAC-implanted mouse as a function of time elapsed after a deuterated glucose injection, for the different approaches here studied. Each panel is spaced by ca. 6 min, for an experimental total of 234 min. Intensities were translated into metabolic concentrations using ^2^H’s natural abundance (~ 10 mM concentration prior to injection in liquid-containing spaces), together with SSFP’s signal attenuations as expected from the scanning parameters and the T1/T2 for each species. Numbers on the bottom indicate to the frames compared in Supporting Information Fig. S3. Notice the initial glucose uptake by the kidney, followed by its accumulation in the tumor before being concentrated in the bladder. Notice as well the HDO gradual signal increase over time throughout the body, as well as the prominent lactate signature at the tumor position.

Figure 3 compares ^2^H images recorded on the same PDAC-implanted mouse using the weighted CSI-SSFP and uniform ME-SSFP experiments, 72 and 79 min after injecting [6,6′-^2^H_2_]-glucose (the entire kinetic set for this mouse is given in Fig. 4). At these time points, the lactate concentration in the tumor is maximal; clear HDO, glucose and lactate contributions are detectable at this time, with the lactate signal prominent and concentrated in the tumor area. Notice that in agreement with the phantom-derived expectations, the weighted ^2^H CSI-SSFP performs noticeably better SNR-wise than its uniform ME-SSFP counterpart, for the HDO, glucose and lactate images.

Figure 4 displays an entire set of DMI results obtained from both kinds of SSFP sequences collected with both uniform and weighted signal averagings. These experiments were executed in an interleaved, time-alternating fashion, to monitor the metabolic transformations in a PDAC-implanted mouse. The enhanced SNR achieved in the ^2^H images by weighted CSI-SSFP is evident for all metabolites. Note that there is no apparent blurring in these data; this may be attributed to the definition losses that these relatively long in vivo abdominal acquisitions undergo over their course as a result of respiration. Supporting Fig. S3 further analyzes these results by summarizing the SNRs observed for each metabolite over this whole set of time-incremented images, when focusing on the tumor region. Notice also in these results, the clear SNR advantages introduced by the weighted acquisition process.

**Figure 5:**
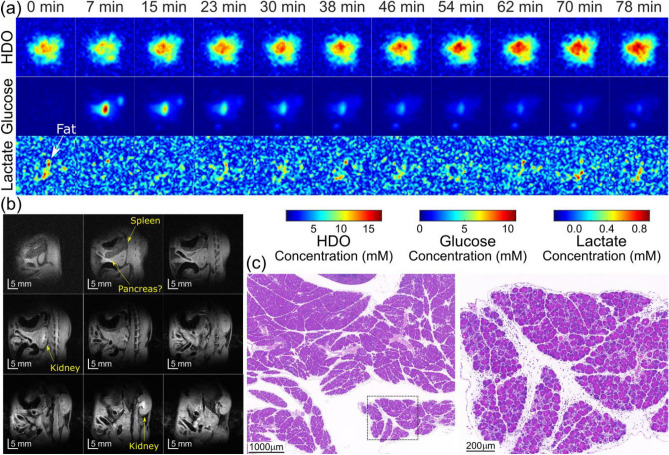
(**a**) DMI images collected on a pancreatitis model as a function of time post deuterated glucose injection. (**b**) Anatomic ^1^H images evidencing the main regions targeted by DMI. (**c**) Histologic sections showing pancreatitis induced in the scanned mouse pancreatitis.

Given the confirmed SNR enhancements achieved with weighted average CSI-SSFP, further investigations were conducted using this sequence to evaluate PDAC, pancreatitis and control cases. As no lactate signal could be detected in the pancreatitis analyses using a relatively moderate weighting (NA = 8), CSI-SSFP’s weighting was increased (NA = 20) to maximize the SNR. Figure 5 illustrates results obtained in this fashion for a prototypical acute pancreatitis mouse. Although sensitivity was high enough to even image small fat signals at ≈1.1 ppm (located very close to the lactate resonance: Supporting Information, Fig. S4), these CSI-SSFP experiments could not evidence the formation of any lactate signal. Neither could non-localized MRS observations observe any changes in the lactate region for these models (data not shown); these changes would have been noticeable despite the presence of a prior resonance at 1.1 ppm, in the form of a time-dependent intensity change.

**Figure 6:**
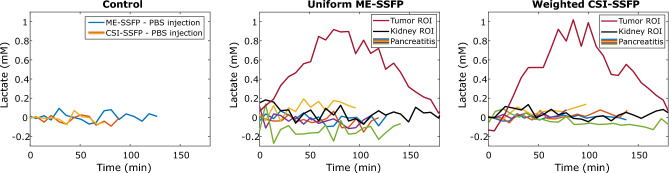
Time courses of various mouse cases for all imaging variants executed in this study, reporting on the integrated signal observed at the [3,3′-^2^H_2_]-lactate’s chemical shift vs time following the initial injection of deuterated glucose. These integrals were taken over similar 20 mm abdominal regions for the control and pancreatitis mice, and over ca. 10 mm diameter tumor/kidney regions in the PDAC case. Notice the clear formation and metabolic kinetic dependence arising for [3,3′-^2^H_2_]-lactate’s in the tumor, vs the baseline levels evidenced by the control mice and the pancreatitis models in all other cases.

Figure 6 summarizes these results, by presenting the lactate kinetics observed by all the methods and on all the different mouse models that were examined in this study, for n = 3 control, n = 10 pancreatitis, and n = 2 representative PDAC cases. The ability of DMI to identify the generation of [3,3′-^2^H_2_]-lactate in the latter, and the absence of any [3,3′-^2^H_2_]-lactate when examining pancreatitis models, is clearly evidenced by these plots—which suggest that in the inflammation cases their levels never reach the 100 µM levels.

## Discussion

The differentiation between pancreatic disfunctions associated to inflammation and to carcinoma is an important open problem in the diagnosis, treatment and prognosis of these diseases. Existing imaging methods—including advanced ones based on PET and on hyperpolarized ^13^C MRI—are challenged by relatively high uncertainties in achieving this discrimination. By depending on both rates of metabolic uptake (like PET) and on rates of metabolic conversion (like hyperpolarized MR), DMI could open a new route to distinguish these two diseases. The present study focused on such problem, using similar model murine systems as were recently analyzed using hyperpolarized methods. The success of these ^13^C MRSI studies was partial: while a variety of pancreatic cancer mice models showed clearly higher lactate/pyruvate ratios arising from the PDAC tumor regions when the latter were relatively large^8,9^, it showed similar rates of pyruvate→lactate conversion (as revealed by the lactate/pyruvate ratio for data recorded within a minute post-injection) for pancreatitis as for mid-sized tumors. DMI could clearly distinguish the two disease models: the uptake of deuterated glucose and its conversion into [3,3′-^2^H_2_]-lactate happened in all PDAC cases analyzed, but it was systematically absent in mice that were subjected to the acute mild pancreatitis model. In an effort to further see if this was related to DMI’s limited sensitivity, a new, alternative ^2^H MRSI acquisition mode was introduced, based on processing phase-encoded FIDs collected under SSFP conditions, using weighted *k-*space sampling and relying on prior chemical shift and field map information. The ensuing CSI-SSFP methods had ≥ 2× better SNR than previous non-linear-fitted SSFP proposals that have been recently discussed—which themselves had ≥ 5× better lactate SNR that kinetic chemical shift imaging methods based on the periodic collection and conventional processing of ^2^H FIDs. Even with these new significant SNR gains, no lactate signal could be observed for the pancreatitis cases. This marks a basic difference between experiments done based on pyruvate and on glucose injections: the former will always originate lactate within a short timescale, whereas the latter will only do so to a significant extent, when driven by the presence of tumors. Supporting Information Fig. S5 presents further evidence for this, by comparing time series of 1D ^2^H MRS data arising from the abdomen of healthy control mice, upon being interrogated by deuterated pyruvate and by deuterated glucose; notice there the buildup in the former of a lactate-derived resonance, which is absent in the latter. Further investigations along these lines are in progress.

A final point worth addressing is the human translation potential opened up by this study. Experiments were here carried out at 15.2 T, thereby benefiting from both the sensitivity brought about by high magnetic fields, and the short acquisition times that at these fields suffice to distinguish various metabolites in ^2^H MRSI. These, however, are not field strengths that are clinically available. Preliminary experiments carried out on a 7 T animal scanner (data not shown) evidence a quadratic decrease in sensitivity for ^2^H MRS with field—ca. a factor of four sensitivity loss from the 15.2 acquisitions. At this lower field, however, also the TR needed to resolve the resonances would have to be nearly doubled, leading to a concurrent halving of the number of scans that can be signal averaged per unit time. In unison these features predict a B_o_^5/2^ field dependence for the sensitivity of these ^2^H MRSI-based tests. When considering their implementation on a clinically available 7 T platform this would demand in turn doubling the in-plane spatial resolution and the acquisition time of the present study; this would still provide diagnostically useful information within ca. 10 min of signal averaging. Reducing the field further to 3 T may already place this study’s approach beyond what is clinically relevant; still, it remains to be seen if adding parallel receiving structures and alternative image processing pipelines, can make up for the lower frequencies. Translational research dealing with these aspects, is currently undergoing.

### Supplementary Information

Supplementary Information.

## Data Availability

The raw data sets and the images generated and analyzed during the current study are available from the corresponding author on request.

## Code availability

The Matlab-based code used for processing the experimental datasets as well as the MRI pulse sequences used in this study, are available at https://www.weizmann.ac.il/chembiophys/Frydman_group/software.
