# Supplementary material for: High-sensitivity deuterium metabolic MRI differentiates acute pancreatitis from pancreatic cancers in murine models
Source: Sci Rep. 2023 Nov 15;13:19998. doi: 10.1038/s41598-023-47301-7 (PMC10652017; doi:10.1038/s41598-023-47301-7)
Supplement: Supplementary file 1 — Supplementary Information. [file 41598_2023_47301_MOESM1_ESM.pdf]

Supporting Information for
High-sensitivity deuterium metabolic MRI differentiates acute pancreatitis from pancreatic cancers in murine models

Elton T. Montrazi,[1] Keren Sasson,[2] Lilach Agemy,[2] Dana C. Peters,[3] Ori Brenner,[4] Avigdor Scherz,[2] Lucio Frydman[1,*]

[1]Department of Chemical and Biological Physics, Weizmann Institute of Science, Rehovot, Israel
[2]Department of Plant and Environmental Science, Weizmann Institute of Science, Rehovot, Israel
[3]Department of Radiology and Biomedical Imaging, Yale School of Medicine, New Haven, USA
[4]Department of Veterinary Resources, Weizmann Institute of Science, Rehovot, Israel
*Email: lucio.frydman@weizmann.ac.il

Supporting Information 1: Acquisition-weighting averaging in DMI

Both k-space acquisition-weighting and apodization have been proposed as ways to improve the SNR of an image collected with a uniform acquisition; i.e., one, in which every k-space point along a phase-encoding axis, *-N/2 ≤ k ≤ N/2-1*, has been sampled by the same number of scans *M*.[1-6] Assuming that the noise in the image is random and uncorrelated, Pakers et al. discussed that the variance, σ^2, that will affect any given pixel [1]

$$\sigma^2 = \frac{\sigma_\psi^2}{N^2} \sum_{k=-N/2}^{N/2-1} \frac{f_k^2}{m_k}, \tag{1}$$

where σ_ψ^2 is the noise variance associated with each measurement, m_k is the number of averages for the kth sampled point, f_k describes the k-space apodization being used, and N is the total number of k-points. In a uniformly sampled, non-apodized acquisition $f_k = 1$ and $m_k = M$ for all $k's$. The noise is then given by

$$\sigma^2 = \frac{\sigma_\psi^2}{MN^2} \sum_{k=-N/2}^{N/2-1} 1 = \frac{\sigma_\psi^2}{MN}. \tag{2}$$

If on the other hand the same acquisition is apodized by a Hanning-window $f_k = 0.5[1 + \cos(4\pi k/N)]$ – which has been shown to represent a good compromise between sensitivity and spatial resolution– the resulting variance will be

$$\sigma^2 = \frac{\sigma_\psi^2}{MN^2} \sum_{k=-N/2}^{N/2-1} f_k^2 = \frac{3\sigma_\psi^2}{8MN}; \tag{3}$$

i.e., a factor of 3/8 smaller. If the weighting is now imparted by applying the same Hanning window coefficients to the number of phase-encoding scans that are actually sampled in k-space while keeping the overall number of scans the same, we have that in equation 1 $m_k = 2Mf_k$ and $\sum m_k = NM$. In such case the variance is

$$\sigma^2 = \frac{\sigma_\psi^2}{MN^2} \sum_{k=-N/2}^{N/2-1} \frac{f_k}{2} = \frac{\sigma_\psi^2}{MN}. \tag{4}$$

which is the same as will arise in the case of the non-weighted, uniform sampling experiment. However, *the signal intensities* in both imaging cases, will be different. This is because the average amplitude of the image as a whole will dependent on the intensity of the sampled signal at the center of the k-space, $k = 0$. In other words, the image brightness will be proportional to the number of averages for m_0. Based on this, an image SNR will be proportional to m_0/σ. In the cases treated above, m_0 = M for the uniform

sampled data (with or without apodization), but it will be $2M$ for the acquisition weighting. Considering this, we obtain that Hanning apodization increases an image SNR by ~1.63x over a plain uniform acquisition, whereas a Hanning weighted acquisition enhances SNR by 2x. Notice however that both of these methods will blur the images: a filter f_k in the k-space is equivalent to a convolution in image space, and for both instances the result is equivalent to weighting-average three adjacent spatial pixels with ratios 1:2:1.[3,4]

In the paper's ME-SSFP experiments, acquisition weighting is achieved by defining a k_y-dependent averaging of the number of scans averaged, according to

$$m(n_y) = (\text{int})\left\{1.5 + (\text{NA} - 1) \cdot 0.5 \cdot \left[1 + \cos\left(\frac{4\pi n_y}{N_y}\right)\right]\right\} \tag{5}$$

where NA is the maximum number of averages set up for the center of the k-space. For the CSI-SSFP experiments the acquisition weighting was applied along the two PE dimensions. The number of scans was in that case set as

$$m(n_x, n_y) = (\text{int})\left\{1.5 + (\text{NA} - 1) \cdot 0.25 \cdot \left[1 + \cos\left(\frac{4\pi n_x}{N_x}\right)\right] \cdot \left[1 + \cos\left(\frac{4\pi n_y}{N_y}\right)\right]\right\}, \tag{6}$$

where $n_x = [-N_x/2 \ \dots \ N_x/2 - 1]$ and $n_y = [-N_y/2 \ \dots \ N_y/2 - 1]$ are the PE indexes along k_x and k_y, with a total number of indexes N_x and N_y respectively. It can be shown that as the spatial encoding is now imparted along two directions, and SNR increase for this 2D acquisition weighted case will be 4x over the SNR arising from a uniformly-sampled, unweighted acquisition. Figures 1c and 1d in the main paper exemplifies the m's arising from Equations (5) and (6), for the 1D ME-SSFP and 2D CSI-SSFP cases, respectively.

Supporting Information 2: Phantom results

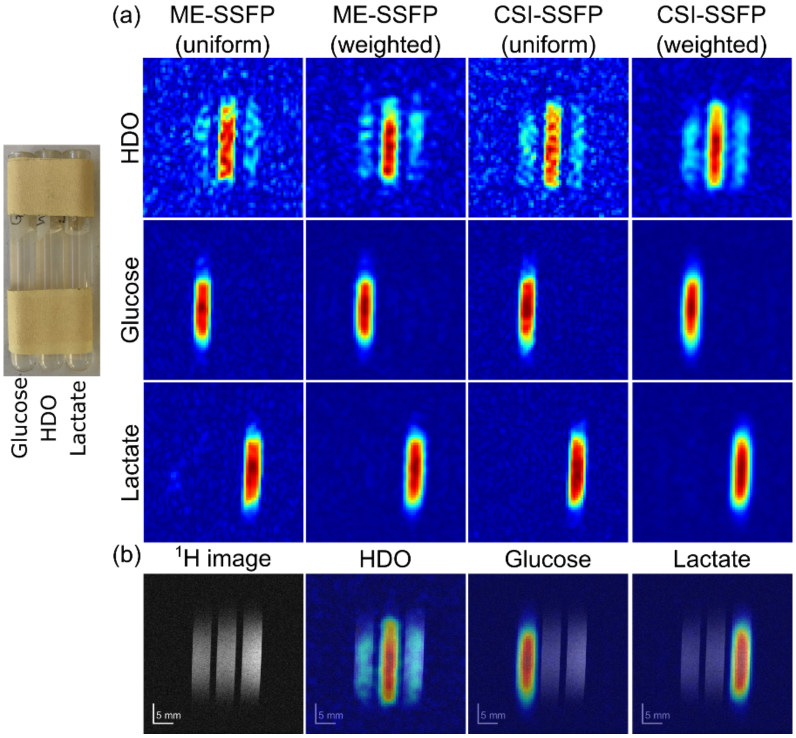

Supporting Figure S1: (a) Comparing the DMI spectroscopic imaging results obtained for the methods listed on the top caption, on a phantom made up by three 5 mm NMR tubes as depicted on the left. (b) DMI images obtained

from the weighted CSI-SSFP experiment, shown overlapped on a ^{1}H reference image. FOVs were in all cases 40x40 mm^{2}.

Supporting Figure S1 compares the ^{2}H spectroscopic images arising from uniform and weighted CSI-SSFP and ME-SSFP experiments; the corresponding SNRs are presented in Table 1 (main paper). These images were obtained on a deuterated phantom, comprising separate tubes for water, glucose, and lactate. The spectral separations were achieved using IDEAL processing pipelines; note that while weighted acquisitions improve notably the SNR, they are also accompanied by some blurring in this well-defined, tube-based phantom data.

Supporting Information 3: Additional histological results

Supporting Figure S2 presents additional representative histological slides evidencing the mild pancreatitis induced by the caerulein-induced treatment.

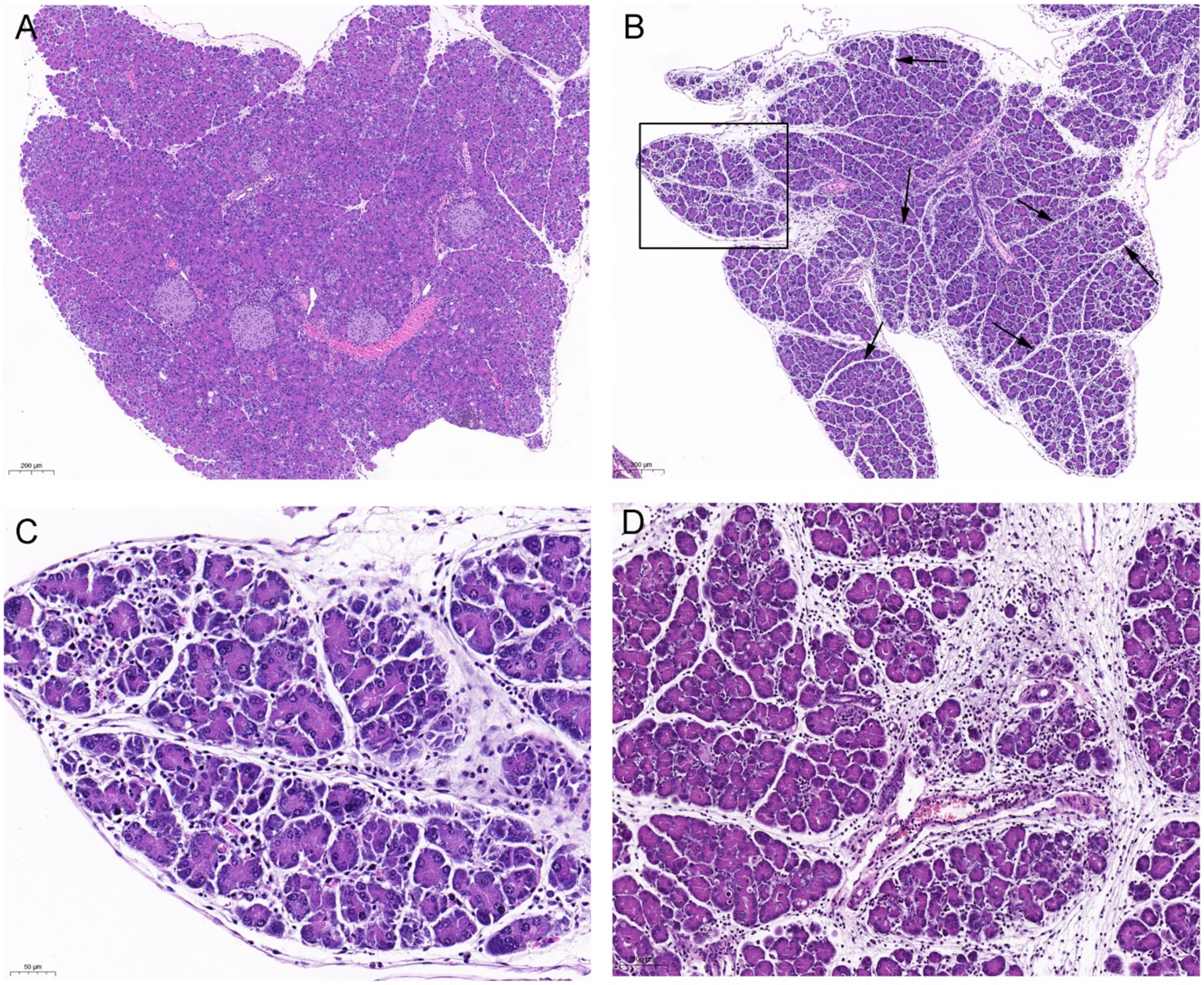

Supporting Figure S2: Representative histological sections illustrating the effects of the caerulein-driven treatment. A: Normal pancreas, showing lobules that are in close contact with each other giving the tissue a compact appearance (x5 magnification; 200 µm marker). B: Pancreatitis-induced pancreas. Notice the expansion of interlobular tissue (arrows) produced by the edema. Inflammatory infiltration is seen as minute dark dots at this magnification (x5 magnification, 200 µm marker). The boxed area is shown in panel C. C: Clear evidence of mild pancreatitis, with mononuclear cells, predominantly lymphocytes, seen as small dark dots within the lobules and in interlobular tissue. (x20 magnification, 50 µm marker). D: Representative field of another mouse showing evidence

of pancreatitis, by the expansion of interstitial tissue caused by the edema and widespread inflammatory infiltration (x10 magnification; 100 µm marker).

Supporting Information 4: SNR summaries for the *in vivo* results

Supporting Figure S3 summarizes the SNRs observed for each metabolite across the entire set of time-incremented images shown in Figure 4 of the main manuscript, with a focus on the tumor region. It is evident that the weighted acquisition process introduces clear SNR advantages. Supporting Table S1 summarizes these SNR improvements for uniform CSI-SSFP, weighted ME-SSFP, and weighted CSI-SSFP acquisitions, taking uniform ME-SSFP experiments as reference standards.

Supporting Table S1: Relative ratios between the SNRs afforded by various approaches for water/glucose/lactate DMI, for the tumor region of the study presented in Figure 4 (main paper).

	HDO	Glucose	Lactate
**Uniform CSI-SSFP / Uniform ME-SSFP**	0.9±0.1	0.8±0.1	1.0±0.1
**Weighted ME-SSFP/ Uniform ME-SSFP**	2.0±0.2	1.3±0.1	1.6±0.2
**Weighted CSI-SSFP / Uniform ME-SSFP**	2.5±0.3	1.4±0.2	2.1±0.2

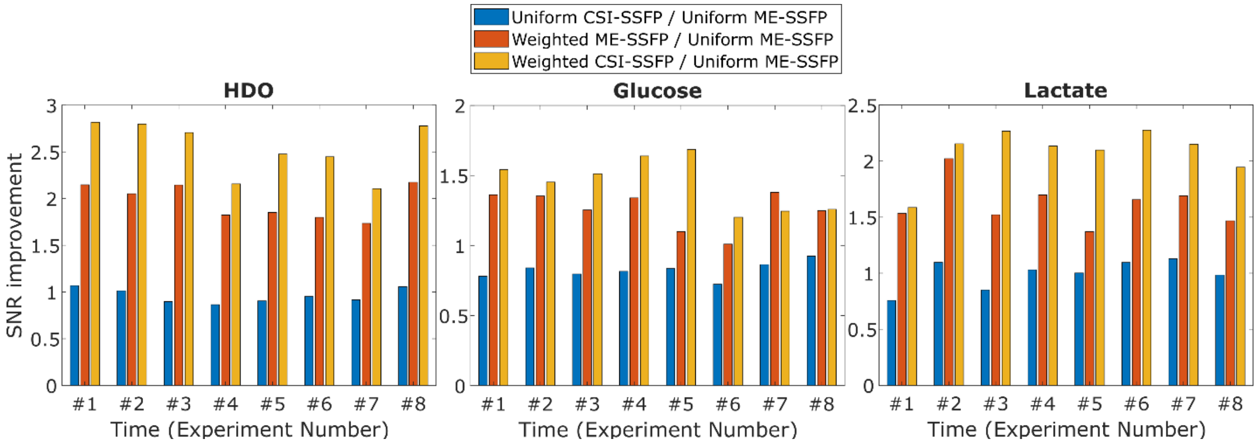

Supporting Figure S3: SNR exhibited by the various DMI sequences assayed, for the tumor region of the same PDAC-implanted animal. Data are taken from Figure 4 of the main paper, and the experiment numbers are associated to the various post-injection times of each experiment in the figure. Notice that given the acquisition times required by these experiments, concentrations are already non-zero in the first data bin.

Supporting Information 5: Fat detection in the ^{2}H MRI experiments

The increased sensitivity achieved by weighted CSI-SSFP enables the detection of fat signals near the ^{2}H coil. Figure 3 illustrates the findings from a pancreatic tumor model. Fat signals are observed in non-localized spectra. In the DMI result before glucose injection, the presence of fat signal is confirmed, and this is further demonstrated by comparing the ^{1}H RARE images collected with fat suppression and without fat suppression. The pre-injection signal in the lactate map remains consistent in all time data.

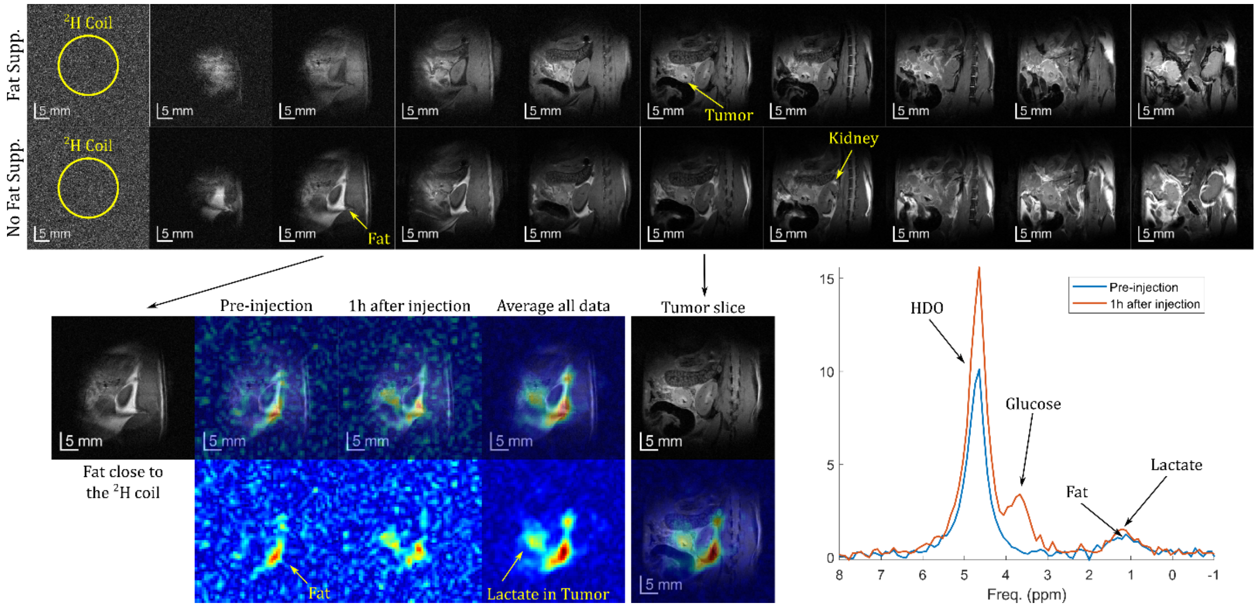

Supporting Figure S4: Top: Anatomical ^{1}H images obtained with and without fat suppression on a PDAC-implanted mouse, highlighting the kidney and the tumor as well as the presence of fat. Also shown on the left are the approximate regions covered by our ^{2}H surface coil. Bottom right: Non-localized in vivo *^{2}H MR abdominal spectra indicating the position of fat signal –very close to where the lactate resonance should appear. Bottom left: ^{2}H CSI-SSFP images overlaid on top of ^{1}H images, revealing the presence of both fat around the kidney and lactate signals within the tumor.*

Supporting Information 6: Deuterated glucose vs deuterated pyruvate experiments on healthy animals, and their differential generation of deuterated lactate

A main difference between pyruvate- and glucose-based experiments is that, even on healthy animals, there will be a significant amount of lactate being generated from pyruvate. This is illustrated in Supporting Figure S5, where ^{2}H MRS non-localized spectra recorded on control animals will show no lactate peak when injected with deuterated glucose, but will evidence the rapid generation of lactate when injected with deuterated pyruvate. This is naturally a disadvantageous aspect when trying to recognize metabolic processes like the Warburg effect, which reflect on an enhanced lactate production.

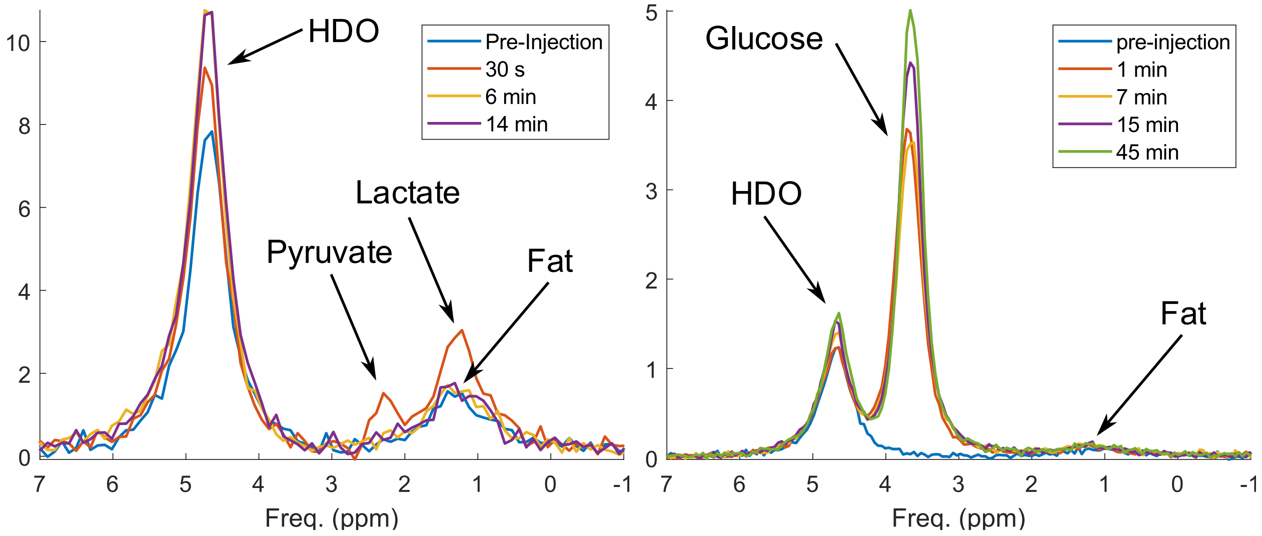

Supporting Figure S5: Comparing non-selective ^2H MRS data collected at the indicated times relative to an intravenous bolus injection, of 3.5 mg of deuterated [$^2H_{3,3'3''}$]-pyruvate (left) and of 15 mg of [$^2H_{6,6'}$]-glucose (right). Both injections were carried out on healthy animals and data were collected using the same CSI-SSFP sequence with the same 20mm diameter coil. Notice the rapid consumption of pyruvate and ensuing transient production of deuterated lactate on the right, vs the lack of lactate on the left. The continuing increase of glucose at long times in the latter case reflects the placement of the coil, which captured the animal's bladder.

References

[1] Parker, D. L., Gullberg, G. T. & Frederick, P. R. Gibbs artifact removal in magnetic resonance imaging. *Med Phys* **14**, 640-645 (1987).

[2] Brooker, H. R., Mareci, T. H. & Mao, J. T. Selective Fourier transform localization. *Magn Reson Med* **5**, 417-433 (1987).

[3] Mareci, T. H. & Brooker, H. R. Essential considerations for spectral localization using indirect gradient encoding of spatial information. *J Magn Reson* **92,** 229–246 (1991).

[4] Hodgkinson, P., Kempharper, R. O. & Hore, P. J. Tailored acquisition in chemical-shift imaging. *J Magn Reson, Series B* **105,** 256–259 (1994).

[5] Adalsteinsson, E., Star-Lack, J., Meyer, C. H. & Spielman, D. M. Reduced spatial side lobes in chemical-shift imaging. *Magn Reson Med* **42**, 314-323 (1999).

[6] Greiser, A. & von Kienlin, M. Efficient k-space sampling by density-weighted phase-encoding. *Magn Reson Med* **50**, 1266-1275 (2003).
